# Supplementary material for: Dog Ecology, Bite Incidence, and Disease Awareness: A Cross-Sectional Survey among a Rabies-Affected Community in the Democratic Republic of the Congo
Source: Vaccines (Basel). 2019 Aug 26;7(3):98. doi: 10.3390/vaccines7030098 (PMC6789516; doi:10.3390/vaccines7030098)
Supplement: Supplementary file 1 [file vaccines-07-00098-s001.pdf]

Supplementary files: Dog Ecology, Bite Incidence, and Disease Awareness: A Cross-Sectional Survey among a Rabies-Affected Community in the Democratic Republic of the Congo

Céline Mbilo, Jean-Baptiste Kabongo, Pati Patient Pyana, Léon Nlonda, Raymond Williams Nzita, Bobo Luntadila, Badivé Badibanga, Jan Hattendorf and Jakob Zinsstag

Table S1. Questions included to assess respondents' knowledge regarding rabies transmission, its symptoms in animals and humans, prevention and control, and practices towards suspected rabid animals and carcasses. No negative scores were given for wrong answers.

Question	Answer	Score correct
1. What is rabies?	Rabies described as a disease	4
	Wrong answer/Unknown	0
2. How is rabies transmitted? (multiple answers possible)	Bite	2
	Scratch	2
	Wrong answer/ Unknown	0
3. Which animals can be infected with rabies?	Dog	2
	Three or more animals mentioned	2
	One or two animals mentioned	1
	Wrong answer /Unknown	0
4. Rabies symptoms in animals[1]	Three or more symptoms mentioned	4
	One or two symptoms mentioned	2
	Wrong answer/ Unknown	0
5. Rabies symptoms in humans[1]	Three or more symptoms mentioned	4
	One or two symptoms mentioned	2
	Wrong answer/ Unknown	0
6. Is rabies preventable?	Yes	4
	No	0
	Unknown	0
7. Methods of prevention and control (multiple answers possible)	Vaccination of humans	1
	Vaccination of animals	1
	Dog population management	1
	Dog bite prevention	1
	Wrong answer / Unknown	0
8. Is rabies curable?	Yes	0
	No	4
	Unknown	0
Overall score knowledge		32
Action taken after exposure (multiple answers possible)	Wound cleaning with water and soap	1
	Post-exposure prophylaxis	1
	Seek medical attention	1
	Anti-tetanus treatment	0.5
	Wrong answer/ Unknown	0
Practice towards suspected rabid animal (multiple answers possible)	Kill the animal	1.5
	Inform veterinary service	2

	Do nothing	0
	Unknown	0
Practice towards carcass of an animal	Inform veterinary service	2
	Take carcass to veterinary service	3.5
	Burry or burn the carcass	1
	Do nothing	0
	Unknown	0

[1] Based on standard case definitions for rabies in the WHO Expert Consultation on Rabies. Third Report [1–3].

References

1. World Health Organization. *WHO Expert Consultation on Rabies. Third Report*; WHO Technical Report Series, No. 1012; World Health Organization: Geneva, Switzerland, 2018.
2. Tepsumethanon, V.; Wilde, H.; Meslin, F.X. Six criteria for rabies diagnosis in living dogs. *J. Med. Asso.c Thai* **2005**, *88*, 419–422.
3. Petersen, B.W.; Rupprecht, C.E. Human Rabies Epidemiology and Diagnosis. In *Non-Flavivirus Encephalitis*; Tkachev, S., Ed.; IntechOpen: London, UK, 2011.
